# A Case of Rhizomelic Chondrodysplasia Punctata in a Neonate

**DOI:** 10.7759/cureus.31702

**Published:** 2022-11-20

**Authors:** Haroon A Javaid, Nader Ashraf, Ramy Mostafa, Nabil Shehata

**Affiliations:** 1 College of Medicine, Alfaisal University, Riyadh, SAU; 2 Department of Pediatrics and Neonatology, Saudi German Hospitals Group, Riyadh, SAU

**Keywords:** congenital disorder, pediatrics & neonatology, peroxisomal disorders, rcdp, rhizomelia

## Abstract

Rhizomelic chondrodysplasia punctata (RCDP) is a rare, multisystem, autosomal recessive, peroxisomal disorder of a family of congenital disorders known as chondrodysplasia calcificans punctate (CCP). RCDP is characterized by disproportionately short extremities (rhizomelia), congenital cataracts, and joint contractures. Dysmorphic facial features include a broad nasal bridge, epicanthus, high-arched palate, dysplastic external ears, and micrognathia. Severe mental retardation with spasticity and seizures may also be present. X-ray of the limbs showed punctate calcifications in cartilage (chondrodysplasia punctata). Genetic testing reveals the severity of phenotype. Treatment is limited to supportive symptomatic relief and prevention of complications. To the best of our knowledge, after searching through PubMed, our case is the first reported case of RCDP in the Middle East.

## Introduction

Rhizomelic chondrodysplasia punctata (RCDP) is a rare, multisystem, autosomal recessive, peroxisomal condition of the family of congenital disorders known as chondrodysplasia calcificans punctate (CCP), which was first recognized by Erich Conradi in 1914. CCP is divided into three primary subtypes: the autosomal dominant Conradi-Hunerman type, the X-linked recessive type, and the autosomal recessive rhizomelic type or RCDP [1]. In Europe and the United States, the autosomal recessive rhizomelic variant of CCP, RCDP, has an estimated incidence of 0.7 and 0.5 cases per 100,000 births, respectively [2]. There are five recognized types of RCDP: 1 (OMIM 215100), 2 (OMIM 222765), 3 (OMIM 600121), 4, and 5 (OMIM 616716), with type 1 being the most common accounting for almost 90% of cases. Associated genes have been identified for each type: PEX7 [3], GNPAT, AGPS [4], FAR1 [5], and PEX5 [6], respectively. 

The genetic subtypes are indistinguishable clinically and there is a range of disease presentations within each type. RCDP is distinguished by disproportionately short stature predominantly in the proximal extremities (rhizomelia); a characteristic facial appearance consisting of a broad nasal bridge, epicanthus, high-arched palate, dysplastic external ears, and micrognathia; punctate calcifications in cartilage with epiphyseal and metaphyseal abnormalities (chondrodysplasia punctata) visible on X-ray, joint deformities (congenital contractures); typical ocular involvement (congenital cataracts); and mental retardation accompanied with stiffness and convulsions. In 1990, it was reported that most children with RCDP die in the first year of life [7], then a study in 2003 reported 90% surviving up to one year and 50% surviving up to six years. However, a more recent study suggested that 75% live to be school-aged. In addition, respiratory dysfunction is the most common cause of death [8]. Although the RCDP population is small, it is growing with unmet medical requirements than are currently recognized, necessitating the need for enhanced diagnostic assistance, increased awareness, and therapeutic development efforts [2]. We aim to discuss the current knowledge on the etiopathogenesis of RCDP as well as the radiological and clinical symptoms of associated diseases. Here, we report a case of RCDP type 1 in a neonate from the Middle East.

## Case presentation

An 11-day-old female Saudi baby was referred from a local hospital in the north of Saudi Arabia for further evaluation and management at our neonatology department. She was born preterm at 36 weeks, via natural spontaneous vaginal delivery. APGAR score was 9 at birth and birthweight was 2.3 kg (low birth weight). Parents were non-consanguineous. 

Upon physical examination, the patient was vitally stable on room air. She was tolerating oral feed with moderate suckling. Her weight was 2.23 kg and her height was 42 cm. Dysmorphic facial features, including low-set ears, depressed nasal bridge, and frontal bossing, were present. A fundoscopic exam showed a hazy view. Arms and thighs appeared short bilaterally with flexion contractures at the knees. The Moro reflex was weak and unequal. There were no episodes of convulsions or abnormal movements. Skin examination showed no abnormal lesions, pallor, jaundice, or signs of cyanosis. Cardiac, chest, and abdominal examinations were otherwise unremarkable.

**Figure 1:**
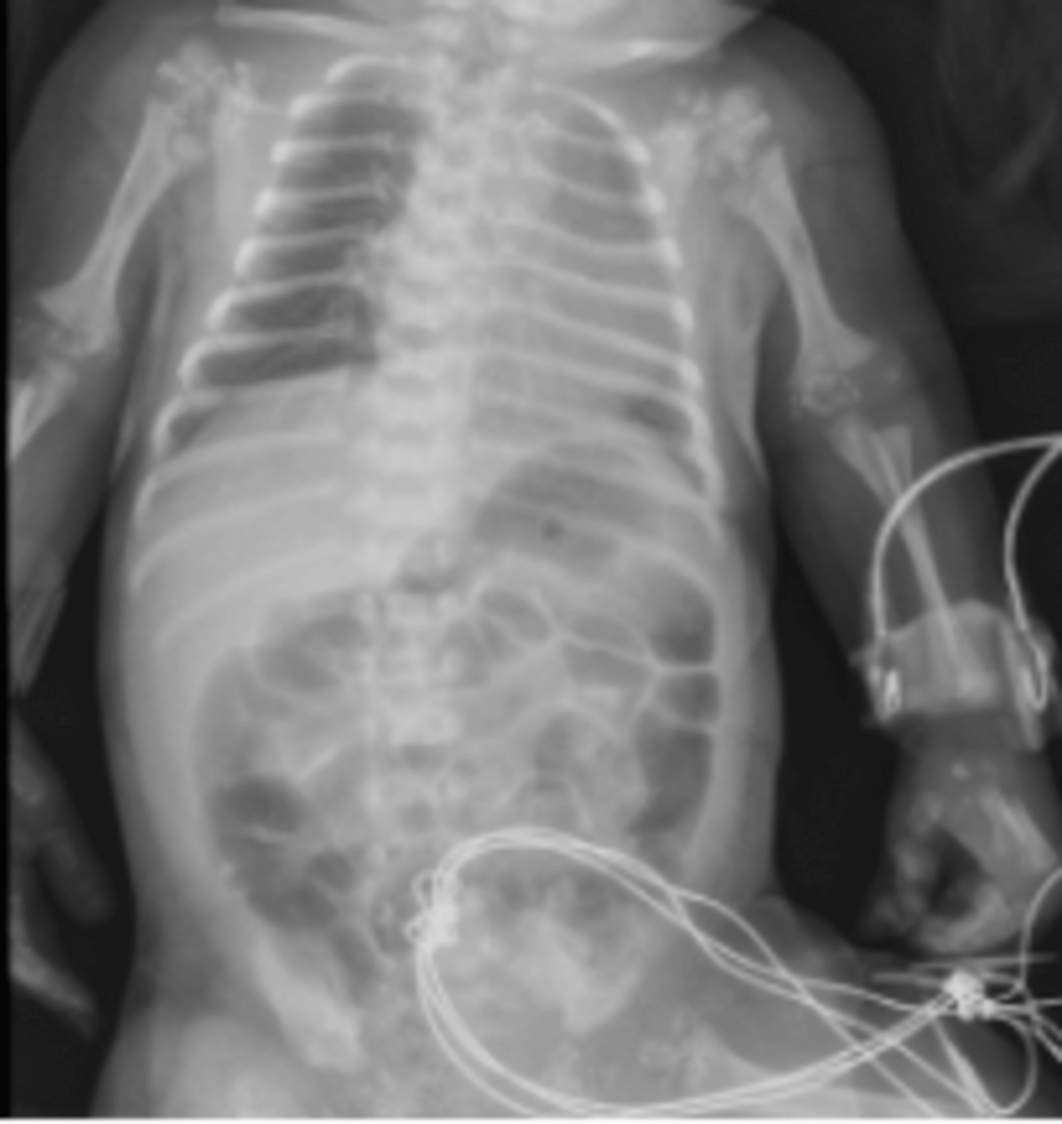
Skeletal Survey (1 of 2): Significant shortening of humeri with mild metaphyseal flaring distally. Discrete punctate cartilaginous calcifications are present at the proximal and distal humeri.

**Figure 2:**
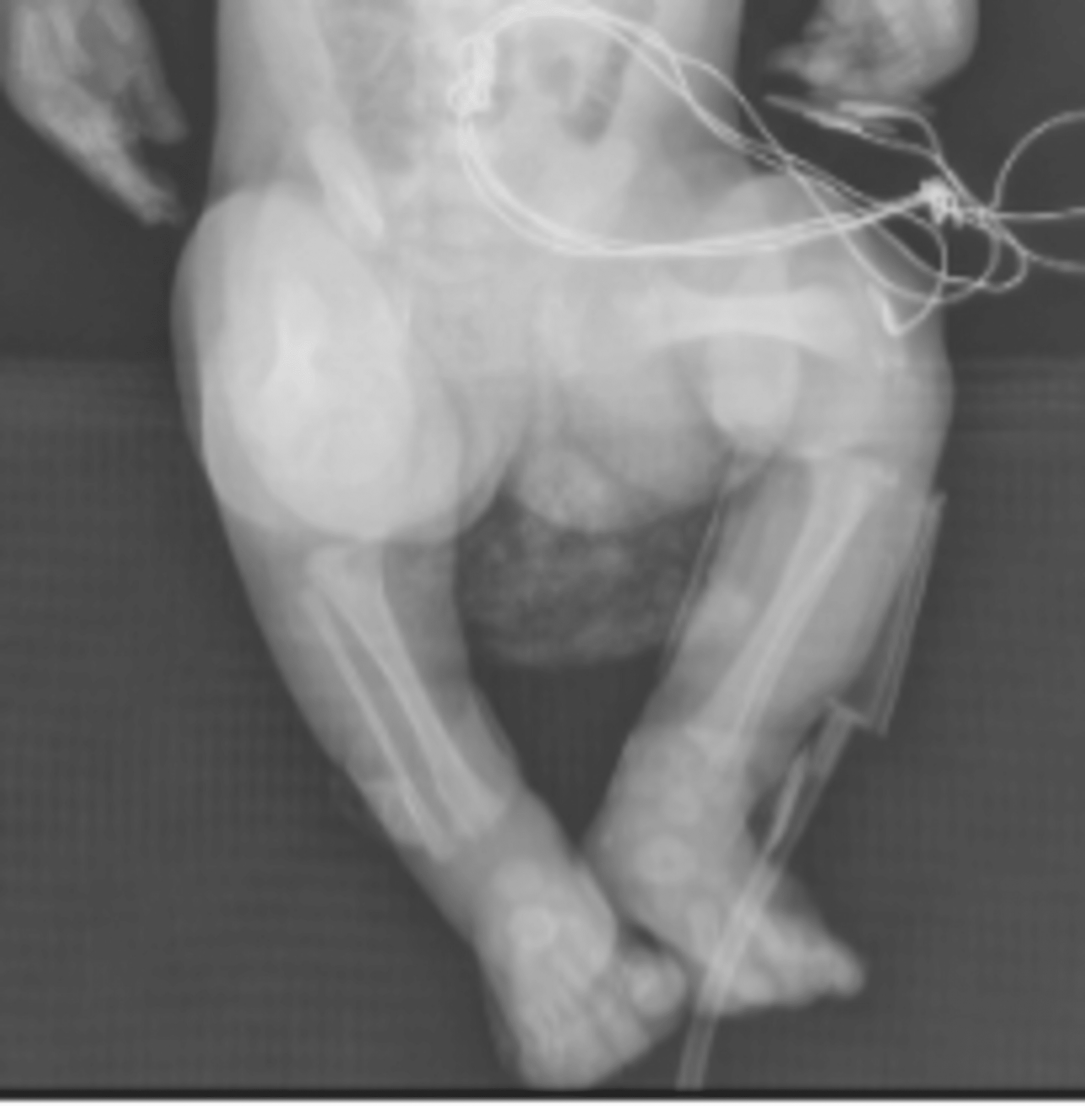
Skeletal Survey (2 of 2): Bilateral shortened femurs with metaphyseal flaring. Discrete punctate cartilaginous calcifications at distal and proximal femurs.

Echocardiography revealed a small atrial septal defect (ASD) with a left to right shunt. Cranial ultrasound with doppler study showed normal findings. A skeletal survey showed significant shortening of both humeri compatible with rhizomelic shortening, with mild metaphyseal flaring of the distal humeri. Discrete punctate cartilaginous calcifications were present at the proximal and distal humeri. Both femurs were shortened with metaphyseal flaring and discrete punctate cartilaginous calcifications were seen at the distal and proximal femurs. Findings were consistent with rhizomelic chondrodysplasia punctata (Figures 1, 2).

**Figure 3:**
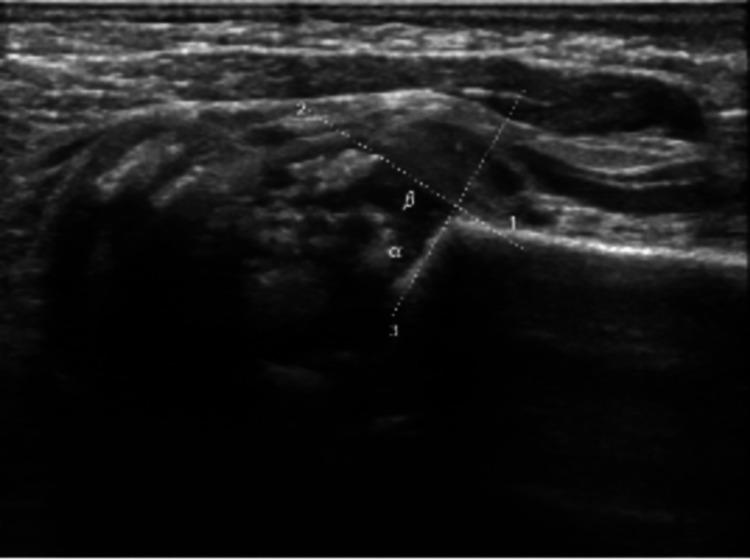
Ultrasound (left hip): alpha angle of 53, beta angle of 56 degrees

**Figure 4:**
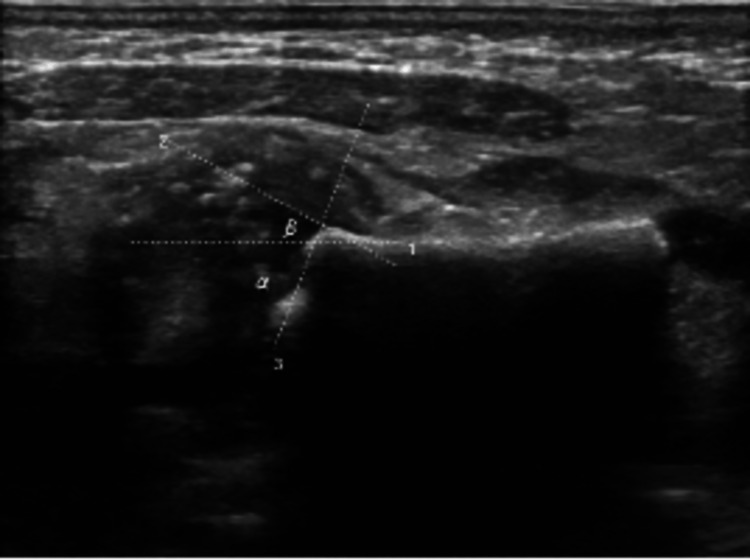
Ultrasound (right hip): alpha angle of 51, beta angle of 52 degrees

Ultrasound findings of both hip joints suggested developmental hip dysplasia (DDH) associated with heterogeneous femoral heads with multiple stippled calcifications. Orthopedic consultation confirmed fetal dysplasia with subluxated hips (DDH type 2). Femoral heads are superiorly displaced from the labrum (Figures 3, 4).

Complete blood count and inflammatory markers were unremarkable. Blood and throat cultures were negative. The metabolic screen for inborn errors of metabolism was negative. Blood chemistry tests were normal. A provisional diagnosis was made via clinical and radiological presentation. Genetic testing results were positive for LDLR and PEX7 genes, confirming the diagnosis of rhizomelic chondrodysplasia punctata. Upon the recommendation of the genetic consultant, very long-chain fatty acid (VLCFA) levels were tested and found to be normal. Pediatric ophthalmology consultation reported bilateral medium-density cataracts. A genetic disease consultation was done.

The patient was provided supportive therapy in the NICU. The patient was placed on IV fluids, IV antibiotics, and partial feeding milk 5ml every two hours along with vitamin D drops. Physiotherapy was arranged for the limb movement and a double pamper was placed for the DDH. She was transferred to a tertiary-care center for cataract surgery. A follow-up for DDH was organized at the orthopedic outpatient clinic. The genetic consultant counseled the parents regarding the condition's diagnosis and prognosis. Upon discharge from NICU, the patient was vitally stable and weighed 2.36 kg. 

## Discussion

Peroxisomes play a vital role in a number of processes in the human body including biosynthesis of ether phospholipids and bile acids, and α- and β-oxidation of fatty acids. Peroxisomal disorders can be categorized into two classes: single peroxisomal enzyme deficiencies and peroxisome biogenesis disorders (PBDs) [9]. In contrast to RCDP2 and RCDP3 which are single peroxisome enzyme deficiencies, RCDP1 is a PBD [10]. There are two types of PBDs, either Zellweger syndrome spectrum (ZSS) or RCDP1. The biochemical changes observed in PBD patients range from an accumulation of substrates broken down by peroxisomes (e.g. VLCFAs, pristanic acid, phytanic acid) to a decrease in products of peroxisomal metabolism (e.g. plasmalogens, cholic and chenodeoxycholic acid) [9]. Although the physiological roles of plasmalogen have been challenging to discern [11], it is seen that neurodevelopmental deficits and age-related occurrences of seizures were milder when plasmalogens were at a higher level in erythrocytes [12]. Disease severity for RCDP1 correlates with erythrocyte plasmalogen levels and has been characterized into non-classic (mild) form and classic (severe) form [13], where plasmalogen levels are almost undetectable. Furthermore, the nonclassical form is characterized mainly by congenital cataracts and chondrodysplasia punctata but, less commonly, can include chondrodysplasia manifesting only as mild epiphyseal changes, variable rhizomelia, and milder intellectual disability and growth restriction than classic RCDP1 [1].

There are various conditions that have comparable punctate cartilaginous alterations including X-linked chondrodysplasia punctata, ZSS, Smith-Lemli-Opitz syndrome, fetal alcohol syndrome, maternal ingestion of certain anticoagulants (dicoumarol or warfarin) in early pregnancy, and even occasionally trisomy 18 [14]. Hence, care must be taken in diagnosing RCDP1 [15]. In addition, RCDP and other peroxisome disorders can present with early-onset seizures [16]. The combination of punctate calcifications, rhizomelia, and biochemical abnormalities (deficient red cell plasmalogens, normal VLCFA, and accumulation of phytanic acid) is pathognomonic for RCDP [12].

Because RCDP1 is a heterogeneous, exceedingly uncommon illness of the fetus and newborn, diagnosis is typically postnatal. Prenatal diagnosis is restricted due to the difficulties in recognizing the causative pathogenic variations during pregnancy. Nonetheless, few reports exist on prenatal diagnosis of RCDP in the literature [17]. Sonography has been known to identify skeletal dysplasia in the antenatal period and raise the suspicion of CCP disorder [1]. Pattern recognition includes disproportionately shortened humeri, indistinct metaphyses, and punctate stippling of the epiphyses. Following a diagnosis, therapy is confined to supportive measures, with an emphasis on symptomatic treatment of symptoms and complications prevention. It is suggested to use genetic testing since it discloses the severity of the phenotype and allows for the early management of clinical symptoms [18]. While there is no cure, an interdisciplinary approach should guide treatment and management and can improve prognosis. Nonetheless, plasmalogen replacement therapy may become available and modify the course of the disease in the future [19]. Furthermore, serial EEG may act as a biomarker for subsequent epilepsy in the RCDP population and guide therapy [20].

## Conclusions

Rhizomelic chondrodysplasia punctata is a rare, heterogeneous, peroxisomal disorder of the neonate. A combination of punctate calcifications, rhizomelia, and biochemical abnormalities (deficient red cell plasmalogens, normal VLCFA, and accumulation of phytanic acid) is pathognomic for the disease. While therapy is mostly symptomatic, interprofessional teamwork is essential, frequently beginning during the prenatal period and lasting into the neonatal period and, on rare occasions, into childhood. To improve patient outcomes and quality of life, practitioners must be educated about best practices for recognizing, diagnosing, and counseling families with RCDP infants.
